# Low Resistance TiO_2_/p-Si Heterojunction for Tandem Solar Cells

**DOI:** 10.3390/ma13122857

**Published:** 2020-06-25

**Authors:** Steponas Ašmontas, Maksimas Anbinderis, Jonas Gradauskas, Remigijus Juškėnas, Konstantinas Leinartas, Andžej Lučun, Algirdas Selskis, Laurynas Staišiūnas, Sandra Stanionytė, Algirdas Sužiedėlis, Aldis Šilėnas, Edmundas Širmulis

**Affiliations:** Center for Physical Sciences and Technology, Savanorių ave. 231, 02300 Vilnius, Lithuania; maksimas.anbinderis@ftmc.lt (M.A.); jonas.gradauskas@ftmc.lt (J.G.); remigijus.juskenas@ftmc.lt (R.J.); konstantinas.leinartas@ftmc.lt (K.L.); andzej.lucun@ftmc.lt (A.L.); algirdas.selskis@ftmc.lt (A.S.); laurynas.staisiunas@ftmc.lt (L.S.); sandra.stanionyte@ftmc.lt (S.S.); algirdas.suziedelis@ftmc.lt (A.S.); aldis.silenas@ftmc.lt (A.Š.); edmundas.sirmulis@ftmc.lt (E.Š.)

**Keywords:** thin films, solar cells, TiO_2_/p-Si heterojunction, atomic layer deposition

## Abstract

Niobium-doped titanium dioxide (Ti_1−x_Nb_x_O_2_) films were grown on *p*-type Si substrates at low temperature (170 °C) using an atomic layer deposition technique. The as-deposited films were amorphous and showed low electrical conductivity. The films became electrically well-conducting and crystallized into the an anatase structure upon reductive post-deposition annealing at 600 °C in an H_2_ atmosphere for 30 min. It was shown that the Ti_0.72_Nb_0_._28_O_2_/*p*^+^-Si heterojunction fabricated on low resistivity silicon (10^−3^ Ω cm) had linear current–voltage characteristic with a specific contact resistivity as low as 23 mΩ·cm^2^. As the resistance dependence on temperature revealed, the current across the Ti_0.72_Nb_0.28_O_2_/*p*^+^-Si heterojunction was mainly determined by the band-to-band charge carrier tunneling through the junction.

## 1. Introduction

The successful development of a monolithic perovskite/silicon tandem solar cell has attracted considerable attention during past few years [1,2,3,4,5,6,7]. The interest in this field is motivated by the rapid development of power conversion efficiency of a perovskite solar cell, from less than 3.8% to above 22% during the last decade [8,9,10,11,12,13,14,15,16]. A monolithically integrated two-terminal (2-T) perovskite/silicon tandem solar cell consists of a top perovskite subcell being deposited onto a bottom silicon subcell. The two subcells are then electrically connected in a series through a recombination layer or a tunnel junction [5]. The tunnel junction consisting of two heavily doped *p*^+^ and *n*^+^ silicon regions was used in the first demonstration of a 2-T perovskite/silicon tandem solar cell [1]. However, the tunnel *p^+^–n^+^* silicon junction can potentially contribute to parasitic optical absorption [7]. Aiming to lower the parasitic optical absorption, Shen et al. proposed to use a recombination junction formed between *p*-Si and atomically deposited TiO_2_, thus enabling to produce a high efficiency monolithic perovskite/silicon tandem solar cell [7].

The electrical properties of TiO_2_ thin films grown by atomic layer deposition (ALD) on crystalline silicon substrates were studied recently [17]. It was found that a heterojunction was formed between the deposited TiO_2_ and silicon substrate demonstrating nonohmic and asymmetric current–voltage characteristics. Usually, TiO_2_ is treated as an n-type semiconductor with a wide bandgap reaching 3.4 eV, 3.2 eV, 3.02 eV and 2.96 eV for amorphous, anatase, rutile and brookite phases, respectively [17,18]. Therefore, nondoped, high quality TiO_2_ has a high resistivity and may serve as an insulator for capacitors [19,20]. The electrical conductivity of nondoped TiO_2_ films can be changed by variations of oxygen concentration during the TiO_2_ reduction [17,18]. The O_2_ deficiency creates defects such as oxygen vacancies, titanium vacancies, and Ti^3+^ and Ti^4+^ interstitials, which may act either as acceptors or donors of electrons [21,22]. TiO_2_ is an amphoteric semiconductor, therefore creation of high conductivity nondoped TiO_2_ films is hindered.

High conductivity semiconductors are needed to produce low resistivity TiO_2_/*p*-Si contact. Furubayashi et al. showed that Nb-doped anatase TiO_2_ film is an optically transparent electrically conducting oxide [23]. The resistivity of TiO_2_ films with a Nb concentration exceeding 6% was less than 2.3 × 10^−4^ Ω·cm at room temperature. Furthermore, Ti_1−x_Nb_x_O_2_ films with *x* ≥ 0.01 showed a metallic behavior. This paper deals with details of fabrication of a highly conducting TiO_2_/*p*-Si heterojunction. Transparent TiO_2_ films were grown by ALD and doped with Nb.

## 2. Materials and Methods

Thin Ti_1−x_Nb_x_O_2_ (mixed titanium niobium oxide) layers were formed on glass, low resistivity *p^+^*-type and low conductivity *p*-type silicon substrates, using a “Fiji F200” atomic layer deposition reactor (Cambridge Nano Tech, Waltham, MA USA). A modular ALD system was used for layer formation in a moderate vacuum. First, glass and silicon substrates (University Wafer, Inc., Boston, MA, USA) were cleaned in ethanol and acetone in an ultrasonic bath for 20 min. Then the silicon surface was thermally oxidized in a quartz tube furnace “SDO-125/3“ (Termotron, Bryansk, USSR) at 1150 °C in air for 3 h. The thickness of the silicon oxide layer was measured by a profilometer “Dektak 6M” (Veeco Metrology LLC, Plainview, NY, USA); it was 150 nm. Round 100-µm diameter holes were formed in SiO_2_ by means of a photolithography technique. Thin Ti_1−x_Nb_x_O_2_ layers were deposited using tetrakis dimethylamido titanium (TDMAT, 99.9%, STREM Chemicals Inc.) and niobium ethoxide (Nb(OEt)_5_, 99.9%, STREM Chemicals Inc.) as precursors for titanium and niobium oxides. Deionized water was used as an oxygen source for both processes. The reactions for both processes are presented below.

Partial surface reactions for TiO_2_:

||Ti-OH + Ti[N(CH_3_)_2_]_4_ → ||Ti-O-Ti -[N(CH_3_)_2_]_3_ + NH(CH_3_)_2_ (1)

||Ti-[N(CH_3_)_2_] + H_2_O → ||Ti-OH + NH(CH_3_)_2_ (2)

Full reaction for TiO_2_:

Ti[N(CH_3_)_2_]_4_ + 2 H_2_O → TiO_2_ + 4 NH(CH_3_)_2_ (3)

Partial surface reactions for Nb_2_O_5_:

||Nb-OH + Nb(OC_2_H_5_)_5_ → ||Nb-O-Nb -(OC_2_H_5_)_4_ + C_2_H_5_OH (4)

||Nb -(OC_2_H_5_) + H_2_O → || Nb-OH + C_2_H_5_OH (5)

Full reaction for Nb_2_O_5_:

2 Nb(OC_2_H_5_)_5_ + 5 H_2_O → Nb_2_O_5_ + 10 C_2_H_5_OH (6)

The reaction chamber was evacuated up to 3 × 10^−2^ mbar before the deposition process. The substrates and the reaction chamber were heated up to 170 °C. A constant flow of 100 sccm of pass-thru and 40 sccm of carrier gas (argon) was used during the deposition process. This kept the reaction chamber at ~0.18 mbar working pressure. To reach the desired vapor pressure, TDMAT and Nb(OEt)_5_ were heated up to 80 °C and 170 °C, respectively. According to the authors of the paper [24], Ti_1−x_Nb_x_O_2_ films deposited at temperatures around 170 °C have maximum electrical conductivity. Deionized water, which was used as an oxidizer, was kept at room temperature. The mixed oxide was formed by inserting a monolayer of niobium oxide after a few consecutive monolayers of titanium oxide (number of TiO_2_ monolayers was selected depending on desired Ti:Nb ratio) and the process was repeated until the desired thickness was achieved. Fabrication of every monolayer consisted of four steps: precursor pulse/purge/water pulse/purge. Timings used for titanium oxide and niobium oxide were 0.2 s/10 s/0.06 s/5 s and 0.2 s/5 s/0.06 s/5 s, respectively. Four hundred monolayers were deposited to achieve an approximately 25 nm-thick coating.

Morphology and composition of Ti_1−x_Nb_x_O_2_ layers were examined by a scanning electron microscope (SEM) “Helios NanoLab 650”(FEI, Hillsboro, OR, USA) equipped with energy dispersive X-ray spectrometer (EDX) “INCAEnergy” (Oxford Instruments, Abingdon, UK). Thin Film ID software (Oxford Instruments) was used to estimate the Ti/Nb ratio with a 3% relative error. A relatively low accelerating voltage (7 kV) was used to achieve higher surface sensitivity. For 5:1 titanium oxide and niobium oxide monolayers deposition the resulting atomic ratio was 72 at.% titanium and 28 at.% niobium. For 10:1 titanium oxide and niobium oxide monolayers deposition the ratio was 84 at.% Ti and 16 at.% Nb.

Crystallographic structure of Ti_1−x_Nb_x_O_2_ layers was studied by X-ray diffraction (XRD) using SmartLab HR-XRD diffractometer (Rigaku, Tokyo, Japan) with an X-ray tube equipped with 9 kW Cu rotating anode. Grazing incidence diffraction geometry was used with the incidence angle of Cu Kα beam set to 0.5° which enabled investigation of thin films and reduced influence of the substrate. The resistivity of Ti_1−x_Nb_x_O_2_ layers was measured using a four-point probe method.

**Figure 1:**
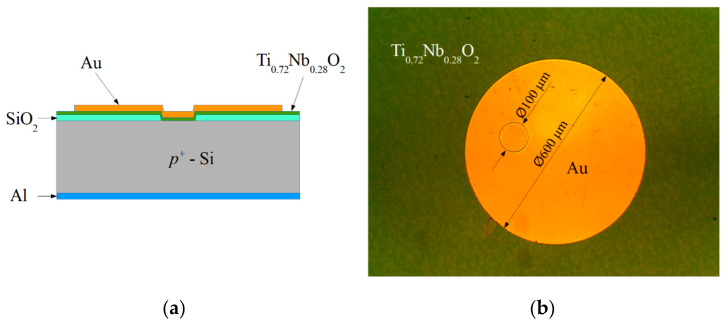
(**a**) Schematic cross-section of the TiO_2_/*p*-Si heterojunction device. (**b**) Microphotograph top view of TiO_2_/*p*-Si (∅ 100 μm) and Au/TiO_2_ (∅ 600 μm) contacts (**b**).

The Si substrates with as-deposited films were divided into two parts, and one part was annealed in the tube furnace at 600 °C in H_2_ atmosphere for 30 min. 500 nm-thick aluminum layer was thermally evaporated using“VAKSIS PVD Vapor-5S_Th” (Vaksis, Ankara, Turkey) on the *p*-type Si immediately after its rear side treatment in HF to remove the unnecessary SiO_2_. To complete the heterojunction devices, ohmic contacts to the *n*-type TiO_2_ were fabricated by thermal evaporation of Ti:Au metal layers with respective thicknesses of 20 nm and: 500 nm onto a photo-resistive mask, and contact patterns were formed using the lift-off technique. Schematic cross-sections of the TiO_2_/*p*-Si heterojunction device and microphotograph of the contacts on the top of TiO_2_ are presented in Figure 1.

Measurements of direct current (DC) current–voltage characteristics of the point-contact TiO_2_/*p*-Si heterojunction were performed using a E5270B Precision IV Analyzer (Keysight Technologies, Inc., Santa Rosa, CA, USA). The point-contact electrical resistance dependence on temperature was measured in a liquid nitrogen vapor atmosphere from 78 K up to 350 K. The temperature of the sample was controlled using K-type Nickel-Chromium/Nickel-Aluminum thermocouple (Thermometrics Corporation, Northridge, CA, USA).

Optical transmission spectra of the Ti_1−x_Nb_x_O_2_ films were measured in the 300–1300 nm wavelength range using AvaSpec ULS2048XL spectrometer and AvaLight-DH-S deuterium-halogen light source (both from Avantes, Apeldoorn, the Netherlands). A 50 ms integration time and an averaging of 100 measured spectra were used for the measurements.

## 3. Results and Discussion

**Figure 2:**
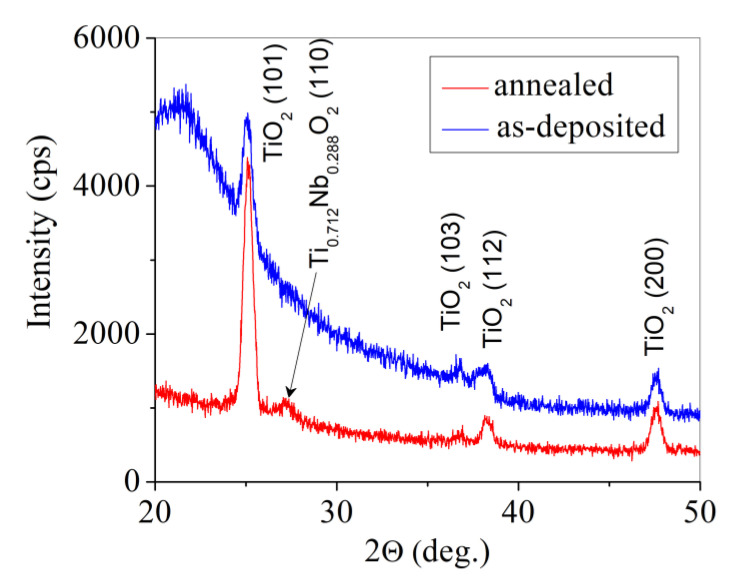
XRD patterns of the Ti_0.72_Nb_0.28_O_2_ film before (dark line) and after annealing in H_2_ atmosphere (red line).

Figure 2 presents the XRD patterns of the Ti_0.72_Nb_0.28_O_2_ film before (as-deposited) and after annealing in an H_2_ atmosphere. It was seen that the as-deposited film had an amorphous structure (a broad feature with maxima at 2Θ angle of about 21.6°) along with a crystalline TiO_2_ of anatase structure. The lattice parameters of anatase tetragonal structure of the film (a = 0.3819 nm and c = 0.9541 nm) were increased in comparison to those presented in ICDD data base card #01-075-2545 (a = 0.3799 nm and c = 0.9509 nm). The increase in lattice parameters should be a result of insertion of Nb ions into crystalline lattice of anatase. After the annealing, the XRD peaks of anatase became sharper as a result of an increase in a crystallite size. The XRD pattern of the annealed film presented one additional peak at 2Θ angle of 27.17°, which could be attributed to niobian rutile Ti_0.712_Nb_0.288_O_2_ (#01-072-7371).

During the annealing, the amorphous phase transformed into an anatase crystalline phase, as XRD measurements confirmed. No characteristic peaks of Nb_2_O_5_ were observed in the Nb-doped TiO_2_ thin film as indicated previously [25].

**Figure 3:**
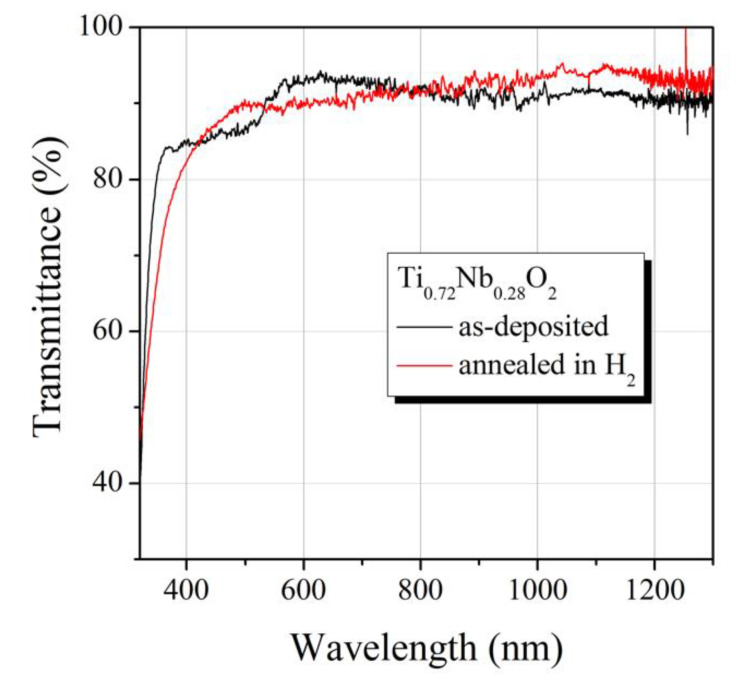
Transmittance of the annealed and as-deposited Ti_0.72_Nb_0.28_O_2_ film on a glass substrate.

Annealing of the Ti_0.72_Nb_0.28_O_2_ films in H_2_ atmosphere also resulted in a substantial decrease of its electrical resistivity from 5.0 × 10^2^ to 1.2 × 10^−3^ Ω cm. In spite of high conductivity, the annealed Ti_0.72_Nb_0.28_O_2_ film was highly transparent in the measured 400–1300 nm spectral region (Figure 3) with more than 93% transmittance within the 800–1000 nm range. Transmittance of the annealed and as-deposited Ti_0.72_Nb_0.28_O_2_ films was significantly higher than that of Ti_0.8_Nb_0.2_O_2_ film [23]. High conductivity and transmittance values suggest the annealed Ti_0.72_Nb_0.28_O_2_ film is a suitable candidate for transparent electrical interconnection for perovskite/silicon tandem solar cells.

**Figure 4:**
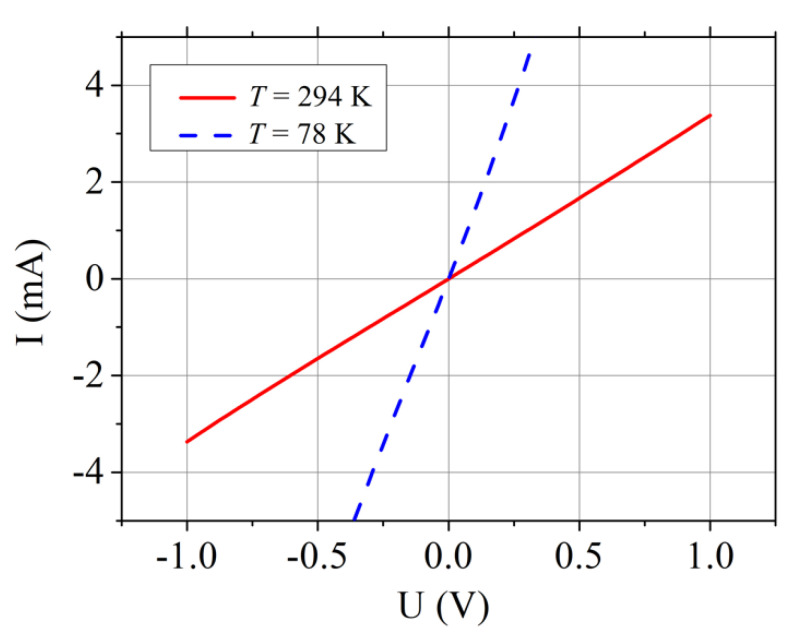
Current–voltage characteristics of TiO_0.72_Nb_0.28_O_2_/*p*^+^-Si heterojunction device formed on the 10^−3^ Ω cm resistivity silicon substrate at room (red solid line) and liquid nitrogen (blue dashed) temperatures.

The current–voltage (I–V) characteristics of TiO_0.72_Nb_0.28_O_2_/*p*^+^-Si heterojunction device on the base of 10^−3^ Ω cm resistivity silicon substrate measured at room and liquid nitrogen temperatures are shown in Figure 4.

**Figure 5:**
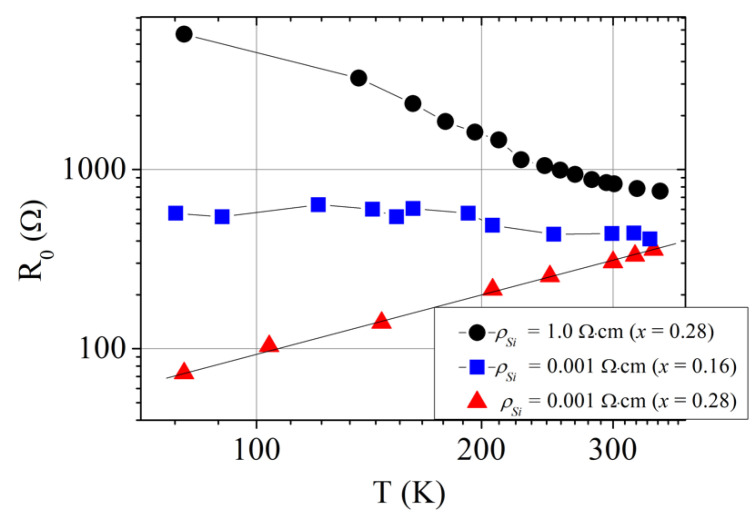
Dependence of the low bias resistance of TiO_1−*x*_Nb_x_ O_2_/*p*^+^-Si heterojunction devices on temperature. The devices differ in the amount of Nb in the TiO_2_ layer (x = 0.28 and 0.16) and in conductivity of the silicon substrate (*ρ*_Si_ = 10^−3^ Ω cm and 1 Ω cm).

It is worth noting that the I–V characteristics were linear; it was another valuable property for the above-mentioned application. The resistance of such TiO_0.72_Nb_0.28_O_2_/*p*^+^-Si heterojunction device decreased as the sample was cooled down. Dependencies resistance-vs.-temperature for TiO_1-x_Nb_x_O_2_/*p*-Si heterojunction devices with different content of x and formed on different Si substrates are depicted in Figure 5. It was seen that the resistance of TiO_0.72_Nb_0.28_O_2_/*p*^+^-Si heterojunction (substrate *ρ*_Si_ = 10^−3^ Ω cm) linearly increased with temperature. Such linear dependence was an inherent feature of the tunnel diode at small bias voltage.

**Figure 6:**
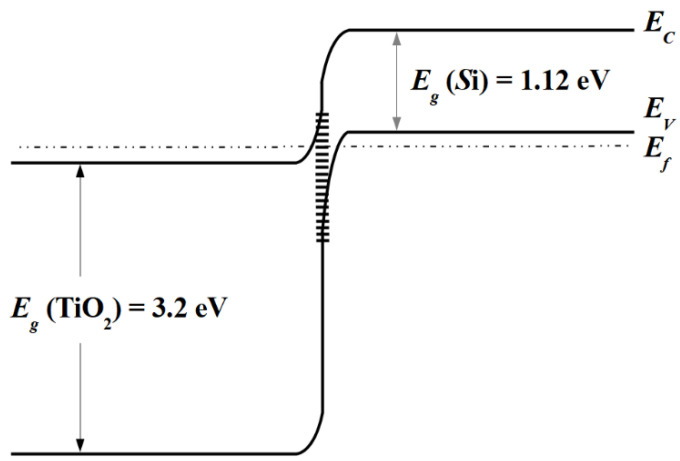
Energy band diagram of *n^+^*–TiO_2_/*p^+^*-Si tunnel heterojunction in an equilibrium condition.

In general, when the interband carrier tunneling takes place, the tunnel current across a *p-n* junction can be expressed as [26,27]:

*I_t_* = *AeU*(*E_v_* − *E_c_*)^2^/4*kT*, (7)

where *A* is a constant, *E_c_* and *E_v_* represent the conduction and the valence band edges, respectively, and *k* is the Boltzmann constant. Expression (7) shows that the resistance of a tunnel *p-n* junction at low bias was a linear function of temperature. The Hall effect measurements indicate that electron density in TiO_0.72_Nb_0.28_O_2_ is 3.5 × 10^21^ cm^−3^ and therefore it can be regarded as a degenerate semiconductor [24,28,29]. Low resistivity *p^+^*-Si (*ρ* = 10^−3^ Ω cm) is also a degenerate semiconductor. The hole concentration determined from Hall effect measurements in *p^+^*-Si was 1.3 × 10^20^ cm^−3^. The energy band diagram of mutually heavily doped *n^+^*-TiO_2_/*p*^+^-Si heterojunction in equilibrium condition is shown in Figure 6. It is seen that electrons from TiO_2_ conduction band could tunnel through the gap to the empty sites of the *p*^+^-Si valence band under a small forward bias. Linear dependence of the resistance on temperature supports the assumption that the interband tunneling current took place in the investigated TiO_0.72_Nb_0.28_O_2_/*p^+^*-Si heterojunction. The specific contact resistivity of TiO_0.72_Nb_0.28_O_2_/*p^+^*-Si heterojunction was 23 mΩ·cm^2^ at room temperature which was better than 30 mΩ·cm^2^ achieved in [7].

As a rule, there are three main components of current in a tunnel diode: the tunnel current (*I_t_)*, the excess current (*I_x_*) and the diffusion current (*I_d_*). The diffusion current is responsible for the current rise under high forward biases [26]. Therefore, *I_d_* should be negligible in comparison with the tunnel current at low bias. The excess current at low bias was mainly determined by the multistep tunneling recombination process via surface states at the TiO_2_/*p^+^*-Si interface [7,30]. Substantial density of localized surface states was determined by a large number of defects, *N_s_* ~ 7.0 × 10^13^ cm^−2^, at the TiO_2_/*p^+^*-Si interface resulting from significant lattice mismatch between the heterojunction components [30]. These interfacial states can facilitate the band to band tunneling and act as generation-recombination centers at all bias voltages [7,31]. As the generation-recombination centers, the interface states had substantial influence on charge transport through TiO_2_/*p^+^*-Si tunnel heterojunction. At reverse bias, every recombination center becomes a source of carrier generation, and high electrical conductivity can be reached by thermally generated carriers [7]. Since the carrier generation–recombination strongly depends on the thermal energy, the multistep tunneling recombination process via surface states resulted in a substantial increase of the heterojunction resistance at lower lattice temperature. Such dependence of the resistance on temperature was observed in TiO_0.72_Nb_0.28_O_2_/*p*-Si heterojunction on low conductivity (*ρ*_Si_ = 1 Ω cm) *p*-type silicon substrate (see Figure 5, black circles). The band-to-band tunneling was impossible in this case, therefore the current through the TiO_0.72_Nb_0.28_ O_2_/*p*-Si heterojunction was mainly determined by the multistep tunneling recombination process via the surface states.

**Figure 7:**
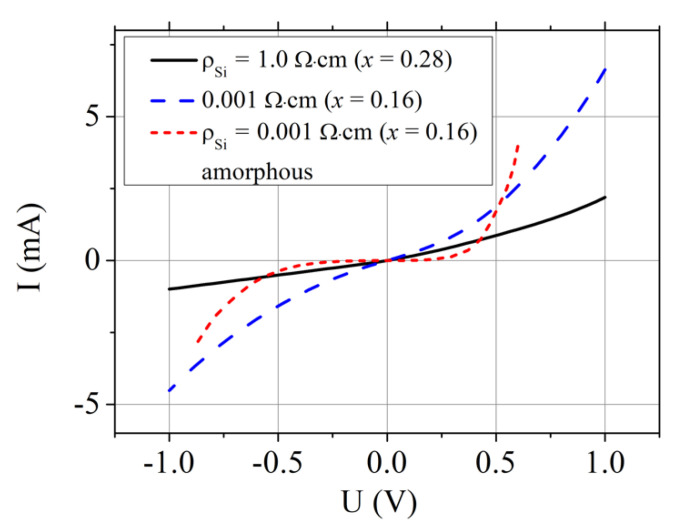
Current–voltage characteristics of TiO_1−x_Nb_x_ O_2_ /*p*-Si heterojunction device measured at room temperature. Solid curve is for x = 0.28 and low conductivity *p-*Si.

I–V characteristic of the TiO_0.72_Nb_0.28_O_2_/*p*-Si heterojunction was not linear (see Figure 7, solid black line) with forward current larger than the reverse one, as observed in other works [7,17,30]. Similar character demonstrated the I–V dependence of the TiO_0.84_Nb_0.16_O_2_/*p^+^*-Si device formed on the low resistivity substrate (Figure 7, blue long-dotted line). Electron density in the TiO_0.84_Nb_0.16_O_2_ film as determined from the Hall effect measurements was 1.9 × 10^21^ cm^−3^. Therefore, the TiO_0.84_Nb_0.16_O_2_ film could be also regarded as a degenerate semiconductor [24,28,29], and the band to band tunnel current could be present in the investigated TiO_0.84_Nb_0.16_O_2_/*p^+^*-Si heterojunction. Since electron density in the TiO_0.84_Nb_0.16_O_2_ was less than in the TiO_0.72_Nb_0.28_O_2_ layer, the tunnel current in TiO_0.84_Nb_0.16_O_2_/*p^+^*-Si heterojunction became of the same order of magnitude as the excess current due to multistep tunneling recombination process via the surface states at the TiO_2_/*p^+^*-Si interface. This consideration was supported by the dependence of the resistance of the TiO_0.72_Nb_0.28_O_2_/*p*-Si heterojunction device on temperature depicted in Figure 5 (blue squares). Very weak temperature dependence of the resistance was an inherent feature of the tunnel current consisting of two components, *I_t_* and *I_x_* [32].

Figure 7 also shows the I–V characteristic of the TiO_0.84_Nb_0.16_O_2_/*p^+^*-Si heterojunction device with as-deposited titanium oxide layer (red short-dotted line). As mentioned above, the as-deposited TiO_0.84_Nb_0.16_O_2_ film had an amorphous structure and therefore its conductivity was low. As a result, the I–V characteristic of the TiO_0.84_Nb_0.16_O_2_/*p*^+^-Si heterojunction device was nonohmic and asymmetric, as observed in other works [7,30].

## 4. Conclusions

Two different (x = 0.16 and x = 0.28) niobium composition containing heavily doped Ti_1-x_Nb_x_O_2_ thin films were ALD-deposited on *p*-type Si substrates. Reductive post-deposition annealing was required to crystallize amorphous titanium dioxide into the anatase structure and to increase its electrical conductivity. The current-voltage characteristic of the TiO_0.72_Nb_0.28_O_2_/*p*^+^-Si heterojunction device is found to be ohmic, and the junction resistance linearly depends on temperature. In this case the current across the heterojunction is mainly stipulated by the interband charge carrier tunneling. When the highly conductive titanium dioxide is deposited on low conductivity (ρ_Si_ = 1 Ω cm) *p*-Si substrate, the current across the TiO_0.72_Nb_0.28_O_2_/*p*-Si heterojunction is mainly determined by the multistep tunneling recombination process via the surface states. The contact resistivity of the TiO_0.72_Nb_0.28_O_2_/*p*-Si heterojunction is higher than that of the TiO_0.72_Nb_0.28_O_2_/*p*^+^-Si heterojunction. The formed titanium dioxide films also demonstrate excellent transparency with absorption less than 10% in the visible region. Therefore, the TiO_0.72_Nb_0.28_O_2_/*p*^+^-Si heterojunction could be a suitable candidate as transparent interconnection in 2-T perovskite/silicon tandem solar cells.

## References

[1] Mailoa J.P., Bailie C.D., Johlin E.C., Hoke E.T., Akey A.J., Nguyen W.H., McGehee M.D., Buonassisi T. (2015). A 2-terminal perovskite/silicon multijunction solar cell enabled by a silicon tunnel junction. Appl. Phys. Lett..

[2] Asadpour R., Chavali R.V.K., Khan M.R., Alam M.A. (2015). Bifacial Si heterojunction-perovskite organic-inorganic tandem to produce highly efficient (*η*_T_* ~33%) solar cell. Appl. Phys. Lett..

[3] Albrecht S., Saliba M., Baena J.P.C., Lang F., Kegelmann L., Mews M., Steier L., Abate A., Rappich J., Korte L. (2015). Monolithic perovskite/silicon-heterojunction tandem solar cells processed at low temperature. Energy Environ. Sci..

[4] Werner J., Weng C.H., Walter A., Fesquet L., Seif J.P., De Wolf S., Niesen B., Ballif C. (2016). Efficient monolithic perovskite/silicon tandem solar cell with cell area > 1 cm^2^. Phys. Chem. Lett..

[5] Werner J., Niesen B., Ballif C. (2017). Perovskite/silicon tandem solar cells: Marriage of convenience or true love story?—An overview. Adv. Matter. Interf..

[6] Zheng J.H., Lau C.F.J., Mehrvarz H., Ma F.J., Jiang Y.J., Deng X.F., Soeriyadi A., Kim J., Zhang M., Hu L. (2018). Large area efficient interface layer free monolithic perovskite/homo-junction-silicon tandem solar cell with over 20% efficiency. Energy Environ. Sci..

[7] Shen H.P., Omelchenko S.T., Jacobs D.A., Yalamanchili S., Wan Y., Yan D., Phang P., Duong T., Wu Y., Yin Y. (2018). In situ recombination junction between p-Si and TiO_2_ enables high-efficiency monolithic perovskite/Si tandem cells. Sci. Adv..

[8] Kojima A., Teshima K., Shirai Y., Miyasaka T. (2009). Organometal halide perovskites as visible-light sensitizers for photovoltaic cells. J. Am. Chem. Soc..

[9] Lee M.M., Teuscher J., Miyasaka T., Murakami T.N., Snaith H.J. (2012). Efficient hybrid solar cells based on meso-superstructured organometal halide perovskites. Science.

[10] Burschka J., Pellet N., Moon S.J., Humphry-Baker R., Gao P., Nazeeruddin M.K., Grätzel M. (2013). Sequential deposition as a route to high-performance perovskite-sensitized solar cells. Nature.

[11] Liu M., Johnston M.B., Snaith H.J. (2013). Efficient planar heterojunction perovskite solar cells by vapour deposition. Nature.

[12] Wojciechowski K., Stranks S.D., Abate A., Sadoughi G., Sadhanala A., Kopidakis N., Rumbles G., Li C.Z., Friend R.H., Jen A.K.Y. (2014). Heterojunction modification for highly efficient organic-inorganic perovskite solar cells. ACS NANO.

[13] Yang B., Dyck O., Poplawsky J., Keum J., Puretzky A., Das S., Ivanov I., Rouleau C., Duscher G., Geohegan D. (2015). Perovskite Solar Cells with Near 100% Internal quantum efficiency based on large single crystalline grains and vertical bulk heterojunctions. J. Am. Chem. Soc..

[14] Li X., Bi D., Yi C., Decoppet J.D., Luo J., Zakeeruddin S.M., Hagfeldt A., Grätzel M. (2016). A vacuum flash-assisted solution process for high-efficiency large-area perovskite solar cells. Science.

[15] Yang W.S., Park B.W., Jung E.H., Jeon N.J., Kim Y.C., Lee D.U., Shin S.S., Seo J., Kim E.K., Noh J.H. (2017). Iodide management in formamidinium-lead-halide-based perovskite layers for efficient solar cells. Science.

[16] Gao X.-X., Xue D.-J., Gao D., Han Q., Ge Q.-Q., Ma J.-Y., Ding J., Zhang W., Zhang B., Feng Y. (2018). High-mobility hydrophobic conjugated polymer as effective interlayer for air-stable efficient perovskite solar cells. Sol. RRL.

[17] Ahiboz D., Nasser H., Aygün E., Bek A., Turan R. (2018). Electrical response of electron selective atomic layer deposited TiO_2-x_ heterocontacts on crystalline silicon substrates. Semicond. Sci. Technol..

[18] Anitha V.C., Banerjee A.N., Joo S.W. (2015). Recent developments in TiO_2_ as n- and p-type transparent semiconductors: Synthesis, modification, properties, and energy-related applications. J. Mater. Sci..

[19] Dueñas S., Castán H., García H., San Andrés E., Toledano-Luque M., Mártil I., González-Díaz G., Kukli K., Uustare T., Aarik J. (2005). A comparative study of the electrical properties of TiO_2_ films grown by high-pressure reactive sputtering and atomic layer deposition. Semicond. Sci. Technol..

[20] Nabatame T., Ohi A., Chikyo T., Kimura M., Yamada H., Ohishi T. (2014). Electrical properties of anatase TiO_2_ films by atomic layer deposition and low annealing temperature. J. Vac. Sci. Technol. B.

[21] Nowotny M.K., Bak T., Nowotny J. (2006). Electrical properties and defect chemistry of TiO_2_ single crystal. I. Electrical conductivity. J. Phys. Chem B.

[22] Bak T., Nowotny J., Nowotny M.K. (2006). Defect disorder of titanium dioxide. J. Phys. Chem. B.

[23] Furubayashi Y., Hitosugi T., Yamamoto Y., Inaba K., Kinoda G., Hirose Y., Shimada T., Hasegawa T. (2005). A transparent metal: Nb–doped anatase TiO_2_. Appl. Phys. Lett..

[24] Niemelä J.-P., Hirose Y., Shigematsu K., Sano M., Hasegawa T., Karppinen M. (2015). Suppressed grain-boundary scattering in atomic layer deposited Nb:TiO_2_ thin films. Appl. Phys. Lett..

[25] Potlog T., Dimitriu P., Dobromir M., Manole A., Luca D. (2015). Nb-doped TiO_2_ thin films for photovoltaic applications. Mater. Des..

[26] Seeger K. (1982). Semiconductor Physics.

[27] Ašmontas S., Gradauskas J., Petkun V., Seliuta D., Sužiedėlis A., Urbelis A. (2005). Hot electron effect in degenerate semiconductor tunnel junction. Acta Phys. Pol..

[28] Nogawa H., Chikamatsu A., Hirose Y., Nakao S., Kumigashira H., Oshima M., Hasegawa T. (2011). Carrier compensation mechanism in heavily Nb-doped anatase Ti_1-x_Nb_x_O_2+__δ_ epitaxial thin films. J. Phys. D Appl. Phys..

[29] Niemelä J.-P., Marin G., Karppinen M. (2017). Titanium dioxide thin films by atomic layer deposition: A review. Semicond. Sci. Technol..

[30] Mostovyi A.I., Brus V.V., Maryanchuk P.D. (2013). Charge transport mechanisms in anisotype n-TiO_2_/p-Si heterostructures. Semiconductors.

[31] Zide J.M.O., Kleiman-Shwarsctein A., Standwitz N.C., Zimmerman J.D., Steenblock-Smith T., Gossard A.C., Forman A., Ivanovskaya A., Stucky G.D. (2006). Increased efficiency in multijunction solar cells through the incorporation of semimetallic ErAs nanoparticles into tunnel junction. Appl. Phys. Lett..

[32] Björk M.T., Schmid H., Bessire C.D., Moselund K.E., Ghoneim H., Karg S., Lörtscher E., Riel H. (2010). Si-InAs heterojunction Esaki tunnel diodes with high current densities. Appl. Phys. Lett..

